# Review: Taurine: A “very essential” amino acid

**Published:** 2012-11-12

**Authors:** Harris Ripps, Wen Shen

**Affiliations:** 1 Departments of Ophthalmology and Visual Science, Anatomy and Cell Biology, Physiology and Biophysics, University of Illinois College of Medicine, Chicago, IL; 2 The Marine Biological Laboratory, Woods Hole, MA; 3 Department of Biomedical Science, College of Medicine, Florida Atlantic University, 777 Glades Road, Boca Raton, FL

## Abstract

Taurine is an organic osmolyte involved in cell volume regulation, and provides a substrate for the formation of bile salts. It plays a role in the modulation of intracellular free calcium concentration, and although it is one of the few amino acids not incorporated into proteins, taurine is one of the most abundant amino acids in the brain, retina, muscle tissue, and organs throughout the body. Taurine serves a wide variety of functions in the central nervous system, from development to cytoprotection, and taurine deficiency is associated with cardiomyopathy, renal dysfunction, developmental abnormalities, and severe damage to retinal neurons. All ocular tissues contain taurine, and quantitative analysis of ocular tissue extracts of the rat eye revealed that taurine was the most abundant amino acid in the retina, vitreous, lens, cornea, iris, and ciliary body. In the retina, taurine is critical for photoreceptor development and acts as a cytoprotectant against stress-related neuronal damage and other pathological conditions. Despite its many functional properties, however, the cellular and biochemical mechanisms mediating the actions of taurine are not fully known. Nevertheless, considering its broad distribution, its many cytoprotective attributes, and its functional significance in cell development, nutrition, and survival, taurine is undoubtedly one of the most essential substances in the body. Interestingly, taurine satisfies many of the criteria considered essential for inclusion in the inventory of neurotransmitters, but evidence of a taurine-specific receptor has yet to be identified in the vertebrate nervous system. In this report, we present a broad overview of the functional properties of taurine, some of the consequences of taurine deficiency, and the results of studies in animal models suggesting that taurine may play a therapeutic role in the management of epilepsy and diabetes.

## Introduction

The impetus for this review dates back more than a few decades, having originated with a curious malady, i.e., the severe headaches that were often suffered by diners who had ingested monosodium glutamate, a common food additive in general use in homes and restaurants. It came to be known by a variety of names, the most common being the “The Chinese Restaurant Syndrome” because of its perhaps excessive use in wonton soup. The cause remained a mystery until 1969, when John Olney and his colleagues unequivocally demonstrated the neurotoxic effects of monosodium glutamate. In an impressive series of papers, they showed that when applied topically or by injection, glutamate and its analogs (aspartate, kainate, N-methyl-d-aspartate [NMDA], α-amino-3-hydroxy-5-methyl-4-isoxazole-propionic acid [AMPA]) were cytotoxic to nerve cells in every part of the central nervous system (CNS) [1-3]. The issue is of more than academic interest, since glutamate-triggered neuronal damage is known to occur when the glutamate concentration of interstitial fluids reaches abnormally high levels as a result of hypoxia, ischemia, or brain trauma.

A striking curiosity was seen when Olney’s studies were extended to the visual system. In the neonatal mouse retina, for example, he reported that a 30 min exposure to parenterally administered glutamate (1 mM) produced a histopathological lesion characterized by swollen cell bodies in the ganglion cell layer, the proximal half of the inner nuclear layer, and extending to the inner plexiform layer. Even after washing and transferring the excised retina to glutamate-free medium, Olney found that the lesion had progressed further, particularly in cells within the inner half of the inner nuclear layer, [2]. It is noteworthy that although the retina had been bathed in glutamate, only the inner layers were seriously affected.

Why had the nerve cells in the distal layers been spared? Neurons and glia have been shown to sequester glutamate via high-affinity uptake systems. These transport mechanisms, regarded as responsible for clearing L-glutamate from the synaptic cleft [4,5] and for terminating the excitatory signal [6], represent the first step in the recycling of the transmitter through the “glutamine cycle” [7,8]. Glutamate uptake undoubtedly plays a cytoprotective role, but it is clearly inadequate to spare the inner retina when exposed to toxic levels of glutamate. Rather, it seems likely that there are one or more endogenous substances that serve to protect the outer retina from the typically severe reaction to glutamate. We suggest that one of the most effective endogenous agents protecting the distal retina from the application of toxic levels of glutamate is the amino acid *taurine*.

### Other cytoprotectants

Before considering further some of the biochemical and physiological features of taurine, as well as the broad range of conditions in which taurine has been shown to be beneficial, we must acknowledge that the retina may be exposed to several other survival-promoting agents under normal conditions. Many that have been shown to be effective, e.g., brain-derived neurotrophic factor (BDNF), ciliary neurotrophic factor (CNTF), and basic fibroblast growth factor (bFGF) were identified and extensively investigated by LaVail and coworkers [9-12]. These and other members of the transforming growth factor-β family help to protect retinal neurons from ischemia, free radical formation, light damage, and related forms of neuronal insult. Although levels of some of these factors are upregulated in response to injury [11,13], these agents, even when applied exogenously, primarily tend to slow the cell death process. Treatment with combinations of antioxidants has also proven to effectively rescue photoreceptors in an animal model (rd1) of retinal degeneration [14], but here too the agents were applied exogenously. We suggest that the high concentration of *endogenous* taurine throughout the retina can better serve the role of neuroprotectant against glutamate-induced excitotoxicity.

### Some Functional Properties

#### A broad-spectrum cytoprotective agent

**Figure 1:**
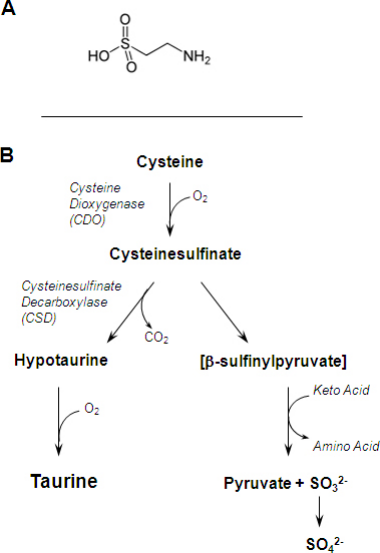
Structure and formation of taurine. **A**: The chemical formula of taurine is C_2_H_7_NO_3_S MW=125.15. **B**: This oversimplified diagram shows the main steps in the conversion of L-cysteine to taurine. The enzyme cysteine dioxygenase (CDO) catalyzes the conversion of L-cysteine to cysteine sulfinate, and the oxidation of hypotaurine (2-aminoethane sulfinate) results in taurine.

Taurine (2-aminoethane- sulfonic acid), an organic osmolyte involved in cell volume regulation, provides a substrate for the formation of bile salts, and plays a role in the modulation of intracellular free calcium concentration [15,16]. Taurine is one of the most abundant amino acids in the brain and spinal cord, leukocytes, heart and muscle cells, the retina, and indeed almost every tissue throughout the body. It was first identified and isolated from the bile of the ox (*Bos* *taurus*), from which it derives its name [17,18]. The chemical structure of taurine, shown in Figure 1A, reveals that it lacks the carboxyl group typical of other amino acids, but does contain a sulfonate group. The major route for the biosynthesis of taurine, shown in Figure 1B is from methionine and cysteine via cysteinesulfinic acid decarboxylase (CSD), and typically requires oxidation of hypotaurine to taurine as the final step [19].

CSD was initially cloned and identified in the liver as the rate-limiting enzyme in the biosynthesis of taurine [20], and was later shown to be present in the kidney as well as the brain, where it is localized in glial cells. CSD levels are very low in cats, as well as humans and other primates, but the ingestion of meat and seafood—or taurine supplements—helps to maintain normal tissue concentrations of taurine. As Sinwell and Gorodischer [21] have shown, there is an increased incidence of pediatric problems in children being raised on the totally vegetarian diets of vegan communities. Aside from the retina, every region of the brain that has been tested contains or takes up taurine; this includes the pineal [22,23], pons medulla [24], hypothalamus [25], striatum [26], and cerebellum [27,28]. At each of these sites, there is evidence of taurine’s ability to ameliorate certain forms of neuropathology.

Because it is one of the few amino acids not used in protein synthesis, taurine is often referred to as a “nonessential” amino acid, or more generously as a “conditionally essential” amino acid. Considering its broad distribution, its many cytoprotective attributes [29,30], and its functional significance in cell development, nutrition, and survival [31,32], these are clearly misnomers. Taurine is undoubtedly one of the most essential substances in the body. Moreover, there is ever-increasing evidence that taurine depletion leads to a wide range of pathological conditions, including severe cardiomyopathy [33], renal dysfunction [34], pancreatic β cell malfunction [35], and loss of retinal photoreceptors [36]. The close relationship between taurine levels and nutritionally induced degeneration is supported further in that taurine supplementation can inhibit light-induced lipid peroxidation, and thereby protect isolated rod outer segments from photic damage [37,38].

There is a long list of diseases that are impacted by taurine, although the precise biochemical mechanism of action is often not entirely clear. A case in point is its role in diabetes. Numerous studies have indicated that taurine plays a significant role in overcoming insulin resistance and other risk factors in animal models of Type 1 and Type 2 diabetes [39-47]. More specifically, taurine administration has been shown to prevent high glucose-induced microangiopathy, i.e., vascular endothelial cell apoptosis [48], and in fructose-fed rats, it has been found to restore glucose metabolizing enzyme activities and improve insulin sensitivity by modifying the postreceptor events of insulin action [49]. The suggestion that nitric oxide (NO) may be implicated in the pathogenesis of diabetes prompted a study to determine whether endogenous NO synthesis or local reactivity to endogenous NO might be impaired in patients with Type 1 insulin-dependent diabetes mellitus [50]. The results showed that either NO- synthase activity is increased or NO sensitivity is decreased in Type 1 patients, a good indication that the L-arginine–NO system is involved in the pathophysiology of diabetes and its sequelae, e.g., diabetic retinopathy. Subsequently, the elevated levels of NO were shown to cause upregulation of the taurine transporter gene and a concomitant increase in taurine uptake in human retinal pigment epithelial cells [51].

Taurine’s effect on renal function [52], particularly as it relates to streptozotocin-induced diabetic animal models, is also noteworthy. As Trachtman et al. (1995) have shown, taurine ameliorates diabetic nephropathy by decreasing lipid peroxidation and lessening the accumulation of advanced glycation end-products in the kidney [39]. However, whether the findings from animal models of diabetes translate to an effective therapy in the management of diabetes in humans is an open question. In this connection, it is important to note that taurine was shown to reduce insulin secretion by β cells in vitro [53]. Moreover, contrary to the results from animal experiments, a study of 20 obese human subjects with a genetic predisposition for Type 2 diabetes demonstrate that taurine supplementation (1.5 g for 8 weeks) had no effect on insulin secretion or sensitivity [54]. In short, these findings do not support the view that dietary supplementation with taurine can be used to prevent the development of Type 2 diabetes. However, it should be noted that this study was clearly too small and of too short a duration to have any clinical significance. Further experimental and clinical studies will be of importance in evaluating taurine’s therapeutic potential in the management of diabetes in humans [45].

Similar issues have clouded the relationship between taurine and epilepsy, although there is little doubt that taurine has antiepileptic activity in experimental animals. The efficacy of taurine has been demonstrated in both naturally occurring and drug-induced epilepsy in cats [55], mice [56], rats [57], and dogs [58], and evidence that taurine blocks dentato-hippocampal synapses, a locus of importance in epileptogenesis, indicates a specific action in epilepsy. Indeed, preliminary experiments in human epileptic subjects confirm the anticonvulsive effect of taurine, but the effects are not robust, nor are they consistent [59]. This may be because taurine does not readily cross the blood-brain barrier, and several taurine analogs that do are currently under investigation for their therapeutic potential [60].

### Taurine in the eye

**Figure 2:**
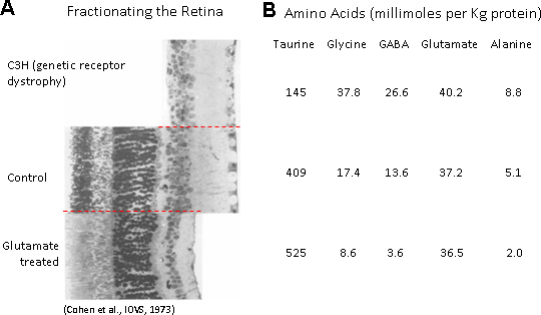
Chemical and genetic fractionation of the retina.** A:** Juxtaposed images of histological sections comparing the retinas of a normal (control) mouse, with one whose inner retina has been damaged by glutamate, and another that was taken from a C3H mouse suffering the loss of the distal retina. **B:** The concentrations of five amino acids in each preparation. The latter values represent the averages from six different groups of dark-adapted animals. (Modified from Cohen et al., 1973, with the permission of the publishers).

It has long been known that all ocular tissues, both neural and nonneural, contain taurine [61,62], prompting a host of studies to identify its cellular distribution [63-66]. Quantitative analysis of whole ocular tissue extracts of the rat eye revealed that taurine was the most abundant amino acid in the retina, vitreous, lens, cornea, iris, and ciliary body [67]. The highest level of taurine was, of course, in the vertebrate retina, and an ingenious experiment involving a judicious selection of normal and diseased mouse retinas enabled Cohen and coworkers [68] to quantify the distribution of taurine and other amino acids across the layers of retinal cells (Figure 2). Note that in the normal (control) retina, taurine exceeds the concentration of each of the other amino acids by tenfold or more, whereas in the photoreceptorless C3H mouse, its concentration is about one-third of its value in the control retina. Note also that destruction of the inner retina by glutamate has little effect on taurine concentration. It is apparent, therefore, that taurine is highly concentrated in the outermost layers of the vertebrate retina. This is consistent with the findings that animals (e.g., cats, monkeys, man) that do not produce adequate levels of taurine experience severe degenerative changes in their photoreceptors and retinal pigment epithelium (RPE) when deprived of dietary taurine [36,69-75].

The selective distribution of taurine within the retinal laminas, as well as in other tissues, is attributable to the presence of both high and low affinity Na^+^- and Cl^-^- dependent taurine transporters [66,76,77]. At the cellular level, the taurine content is determined primarily by the sum of three processes: (i) its synthesis from methionine/cysteine, (ii) its active uptake by the taurine transporter, and (iii) its release via a volume-sensitive leak pathway [78]. The principal transport protein is the saturable, high-affinity TauT transporter (K_m_=18 μM), a member of the neurotransmitter transporter family that includes the transporters for serotonin, creatine, and gamma amino-butyric acid (GABA) [79]. All members of this family have 12 membrane-spanning helices, with the N- and C-terminal ends exposed to the cytosol [80]. The cytosolic domains contain several highly conserved serine, tyrosine, and threonine residues that provide sites for phosphorylation. In terms of its stoichiometry, the active uptake of one molecule of taurine requires two to three sodium ions and one chloride ion [78], and only guanidinoethyl sulfonate (GES) and other close analogs of taurine, e.g., β-alanine, GABA, are inhibitors of taurine uptake. Interestingly, both a GABA transporter and a taurine transporter are active at apical membrane vesicles from bovine RPE; they both require Na^+^ and Cl^-^ and exhibit a similar stoichiometry. An analysis of taurine uptake at this site showed that uptake was severely depressed in the presence of GABA, and conversely, GABA uptake was suppressed by the presence of taurine [81].

Depletion of taurine in rats treated with GES leads to a marked and progressive reduction in the amplitude of the electroretinogram [82] and severe degenerative changes in photoreceptors and the RPE [83], effects that can be reversed by intravenous infusion of taurine [84]. A precipitous loss of taurine is also seen after genetic disruption of TauT in mice. In this model, there is severe photoreceptor degeneration 2–4 weeks after birth, and this spreads to the inner retinal neurons after 4 weeks [85]. Clearly, endogenous taurine is crucial for preventing retinal neurodegeneration. Findings such as these, although difficult to interpret precisely, add to an appreciation of the importance of taurine in the cell biology of the retina.

### Taurine and cytoprotection

Photoreceptors are considerably richer in taurine than other retinal neurons, but all retinal cells from the outer and inner nuclear layers to the ganglion cell layer, and seemingly the radial glia (Müller cells) as well [86], take up taurine from the extracellular milieu [66,87-91]. Therefore, it is not surprising that depleting endogenous taurine by the genetic knockout of TauT or by blocking the taurine transporter with GES has been shown to cause ganglion cell loss, along with degenerative changes in the distal retina [75,85]. It is apparent that taurine serves a neuroprotective role in ganglion cells, as well as in photoreceptors. On the other hand, it was surprising to learn that the early in vivo experiments on the active transport of taurine through the frog RPE showed the main flux to be in the retina to choroid direction [92]. However, the ship was righted with evidence of bidirectional transport [93], and the demonstration of taurine transport from the blood to retina direction [94]. In addition, passive diffusion of such a small molecule as taurine would allow it to traverse the plasma membrane of retinal cells without the aid of an active transport mechanism, and there is experimental evidence that both path length and matrix components (collagen and elastic tissue) influence the diffusion of taurine across human and bovine tissues comprised of Bruch’s membrane–choroid [95].

Perhaps the most exhaustive body of experimental work on the neuroprotective properties of taurine was performed by Wu and colleagues [29,30,96-98]. These innovative studies provide convincing evidence that there are several avenues by which taurine exerts its protective role. Using primary neuronal cultures from the fetal rat brain, these researchers showed that taurine suppresses glutamate-induced toxicity through several pathways: (i) it inhibits calcium influx through L-, N- and P/Q-type voltage-gated calcium channels, (ii) it prevents the downregulation of Bcl-2 and the upregulation of Bax, the protein products of which otherwise would translocate to the mitochondria and result in the release of the highly toxic cytochrome C (cyC), (iii) it protects neurons from oxidative stress, and (iv) it inhibits glutamate-induced calpain activation, thereby preventing the cleavage of Bcl-2 (see also [99]).

There is obviously a broad array of mechanisms by which taurine serves its cytoprotective role, but the molecular identity of a taurine-selective receptor remains a mystery. Several studies have implicated a metabotropic GABA_B_-binding site as mediating the action of taurine, particularly in the brain regions of the mouse and rat [100,101], as well as in the mammalian retina [75]. However, the pathway linking the GABA_B_ receptor to its physiologic action has yet to be identified, and there is a high level of uncertainty regarding the existence or nature of a taurine-specific receptor (see below).

### An experimental study

One of the many experiments demonstrating the cytoprotective action of taurine is based on the now well-established fact that when cells die, they tend to generate toxic substances. These toxins can pass through gap junctions to kill their neighbors, a process referred to as “bystander” cell death [102-104]. Because RPE cells are extensively interconnected via gap junctions [105], a human RPE cell line (ARPE-19) expressing Cx43 and Cx46 was chosen to conduct an experiment that directly tested the efficacy of taurine in the prevention of cell death [106]. Using a very fine blade, a small incision was made in the monolayer of ARPE-19 cells, and a solution of the potent cytotoxin cyC was applied to the site of the cut. Since cyC (molecular mass ~12 kDa) cannot pass through the cell wall, nor can it traverse gap junctions, entry was confined to the narrow row of injured cells. However, not only did cyC induce the death of cells along the scrape, but it also caused apoptosis in cells remote from the site of injury. In contrast, when the cells were preincubated in taurine, or the gap junctions were blocked with octanol, cell death was confined to those cells that were injured by the scrape. To ensure that the taurine effect was not due to the blockage of gap junctions, voltage clamp recordings from electrically coupled *Xenopus* oocytes transfected with Cx43 showed that junctional communication was not affected by taurine [106].

We should stress that experimentally induced cell death by cyC (as used in the foregoing study) simply bypasses the usual mitochondrial pathway to apoptosis. In more physiological circumstances, pathological conditions often lead to mitochondrial dysfunction, triggering the release of cyC, activation of a downstream caspase cascade, and eventual nuclear disruption. How taurine interferes with this process is unclear, although the results of experiments by Takatani et al. [107] suggest that taurine inhibits apoptosis by preventing the formation of the Apaf-1/caspase 9 apoptosome, a key stage in the mitochondrial pathway to cell death. However, this finding has not been independently confirmed, nor as we have already mentioned is it likely to be its sole mode of action.

### Its role in development

In addition to its protective and therapeutic actions, taurine has proven essential for normal development [85,108], and the genetic TauT knockout mouse has been valuable in this regard. Without appropriate taurine uptake, cell degeneration is inevitable, and this mouse line experiences birth defects in their mitochondria, and in myocardial and skeletal muscle development, e.g., increased ventricular wall thickness and cardiac atrophy.

Taurine also plays a critical role in brain development. Taurine deficiency leads to a delay in cell differentiation and migration in cerebellum, pyramidal cells, and visual cortex in cats and monkeys [109-113]. Moreover, Hernandez-Benitez et al. [114] have shown that taurine promotes neural development not only in embryonic brain, but also in adult brain regions. Of particular interest is the fact that within the subventricular zone of the cultured adult mouse brain, taurine activates stem cells and neural precursor cells to differentiate into neurons rather than astrocytes. The subventricular zone is one of the few regions in the brain in which neurogenesis continues throughout adulthood, and the cells from this region can proliferate and migrate via the rostral migratory stream to the olfactory bulb where they differentiate into neurons [115]. Considering the high taurine content in the adult olfactory bulb, it is likely that taurine is an important factor for neurogenesis. It should also be noted that the actions of taurine on adult subventricular stem cells and progenitor cells are not mimicked by glycine, GABA, or alanine [114].

The importance of taurine in retinal development was revealed in many of the earlier studies in which endogenous taurine was depleted by the taurine transport inhibitor GES, or by feeding mothers and their newborn taurine-free diets. The findings showed that taurine deficiency during the early stages of retinal development leads to impaired photoreceptor development, loss of ganglion cell axons, a higher frequency of fetal resorption, and stillbirth [109,110,116-119]. Perhaps even more relevant are the striking results from the Cepko laboratory, where it was shown that taurine stimulates rod development when added to media containing rat retinal cultures [120]. Interestingly, taurine uptake could be blocked without inhibiting its ability to stimulate rod production, evidence that the mechanism of action is neither osmoregulatory nor nutritive. Subsequent studies have implicated the ligand-gated glycine α2 receptor in photoreceptor development [121], since mice with targeted deletion of this receptor no longer experienced proper normal photoreceptor development. However, the spotlight focused once again on taurine when a genome-wide analysis identified a noncoding RNA expressed in the developing retina, taurine upregulated gene 1, and that its knockdown with RNA interference resulted in malformed or nonexistent photoreceptor outer segments [122].

Further evidence for the involvement of taurine in retinal development was provided in a recent study showing that under defined culture conditions, taurine (and certain growth factors) can efficiently promote the in vitro generation of putative rod and cone photoreceptors from mouse, monkey, and human embryonic stem cells [123]. The suggestion that taurine’s ability to promote photoreceptor development may be mediated by GlyRα2 subunit-containing glycine receptors [124] is apparently at odds with the evidence that neither the addition of glycine nor GABA to the media had the same effect as taurine [125].

### Taurine and oxidative stress

It has become increasingly apparent that oxidative stress plays a major role in a broad range of human diseases. The overproduction of reactive oxygen specie and the body’s inability to stem the accumulation of highly reactive free radicals have been implicated in cardiovascular disease [126], diabetes-induced renal injury [127], inflammatory disease [128], light-induced lipid peroxidation in photoreceptors [38], reperfusion injury [129], and several of the major disorders of the CNS [130,131]. In each case, taurine, by virtue of its antioxidant activity, has been shown to play a crucial role as a cytoprotectant and in the attenuation of apoptosis. Despite this diversity of pathophysiology in so varied a group of seemingly unrelated disorders, there is a growing consensus that oxidative stress is linked to mitochondrial dysfunction [127,130-133], and that the beneficial effects of taurine are a result of its antioxidant properties [126,128,129], as well as its ability to improve mitochondrial function by stabilizing the electron transport chain and inhibiting the generation of reactive oxygen species [134,135].

This mode of action has been described by Schaffer and coworkers [135] in cases of diabetes. They find that in this condition, there occurs a decline in the levels of endogenous taurine, and suggest that this taurine deficiency reduces the expression of the respiratory chain components required for normal translation of mitochondrial-encoded proteins. They propose that the dysfunctional respiratory chain accumulates electron donors, thereby diverting electrons from the respiratory chain to oxygen, and forming superoxide anion in the process. Increasing taurine levels restores respiratory chain activity and increases the synthesis of ATP at the expense of superoxide anion production.

### Taurine and neurotransmission

Perhaps the most enigmatic question regarding taurine is whether it is a neurotransmitter. The structural resemblance between γ-aminobutyric acid and taurine, the similar distributions of these amino acids and their synthesizing enzymes in various regions of the brain, and the evidence that taurine, when applied to CNS neurons, exerts an inhibitory effect on their firing rate [136] have all contributed to the view that taurine is indeed a neurotransmitter. Adding to this is the fact that there is a rapid calcium-dependent efflux of taurine after electrical stimulation of cortical slices of rat brain, and the presence of uptake mechanisms to terminate its action [137-139]. Nevertheless, the issue is far from resolved, and the effects of taurine on the responses of retinal neurons have served to highlight some of the difficulties.

In their initial studies on the action of taurine on neuronal pathways in the rabbit retina, Cunningham and Miller [140] showed that taurine was able to separate the ‘On’ and ‘Off’ channels of the parallel pathways identified in recordings of the electroretinogram, the proximal negative response of amacrine cells [141], and the spontaneous activity of ganglion cells. Without detailing the findings in this paper, it is noteworthy that application of 20 μM strychnine blocked the neuronal effects of taurine, suggesting that taurine was acting on receptors that were also responsive to glycine. Subsequent studies by these authors on the actions of both of these agents revealed that the same concentrations of either amino acid had similar effects on intra- and extracellular recordings from retinal neurons and Müller (glial) cells [142,143]. The fact that this array of responses to both taurine and glycine were blocked by strychnine suggests that a single glycinergic receptor may be sensitive to both agents. However, there is some evidence to the contrary. For example, the inhibitory actions of both glycine and taurine on the frog spinal cord are blocked by strychnine, but the hyperpolarizing effect of taurine could be blocked by a strychnine concentration of 100 μM, which had no effect on the response to glycine [144]. In addition, the taurine antagonist TAG (6-aminomethyl-3-methyl-4H,1,2,4-benzothiadiazine-1,1-dioxide) blocks spinal cord depolarization without affecting the similar response to glycine [145]. Thus, although the actions of glycine and taurine overlap at similar receptors, there is reason to suspect that the receptor populations are not the same [146].

A similar situation arose with the inhibitory neurotransmitter GABA, another ω-amino acid whose molecular structure is strikingly similar to that of glycine and taurine. Once again it was difficult to clearly distinguish between their neuronal actions. Electrical stimulation significantly enhanced both the formation and efflux of GABA and taurine in isolated synaptosomes from the mouse brain, and the kinetic parameters for their high affinity uptake were almost identical [147]. Moreover, equivalent amounts of taurine and GABA depressed the firing rate of brainstem neurons almost equally [148], and similar specific, carrier-mediated transport systems are known to operate at brain cell membranes [149,150]. However, unlike the findings with glycine, there are significant differences between taurine and GABA. Both in retina and isolated synaptosomes, strychnine suppressed the action of taurine but not that of GABA, whereas the GABA antagonist bicuculline had no effect on the inhibitory action of taurine, but blocked the depressant action of GABA. In sum, these observations suggest that taurine and GABA are acting on different receptors, and thus, there is no convincing evidence that the electrophysiological actions of taurine are mediated via binding to an ionotropic GABA receptor.

### Criteria that define a neurotransmitter

Uncertainties as to whether a molecule is a neurotransmitter have led to the establishment of various criteria (some more essential than others) for inclusion. These are as follows:

(i) Evidence that the substance, together with the enzymes and related chemical machinery required for its synthesis are present within the presynaptic neurons;

(ii) Evidence that the substance is released by a calcium-dependent mechanism in response to presynaptic depolarization, and that it exerts an effect on postsynaptic cells;

(iii) The presence of a mechanism to terminate the action of the transmitter (e.g.., degradation, high-affinity uptake), and the availability of a relatively specific antagonist; and

(iv) The presence on postsynaptic cells of a receptor that specifically binds the putative neurotransmitter.

Studies too numerous to cite here have shown that agents such as GABA, glutamate, acetylcholine, and glycine satisfy these criteria, and studies already cited show that taurine satisfies all but one of the above criteria. Thus, although taurine is released after electrical stimulation, and at physiologic concentrations it exerts a powerful inhibitory effect on the bioelectric activity of the retina and on synaptic transmission in retinotectal pathways, the one crucial criterion that has not yet been met is *the presence of a taurine-specific receptor on postsynaptic cells*.

There is no shortage of publications claiming to have detected one or more putative taurine receptors. Results obtained by Kudo et al. [151] on the effects of taurine in the frog spinal cord were interpreted as revealing two taurine receptor subtypes. This conclusion was based on their observation that the application of 10 mM taurine caused a biphasic response consisting of a hyperpolarization followed by a slow onset depolarization. The former was selectively depressed by low concentrations of bicuculline that had no significant effect on the antagonizing action of GABA, whereas the hyperpolarizing component was selectively reduced by a strychnine concentration that had no effect on the response to glycine. Clearly these findings are highly suggestive, but cannot be considered definitive evidence of the presence of taurine-specific receptors. Other studies purporting to have detected taurine receptors in rabbit brain [152] and in RPE cells in culture [153] have been similarly inconclusive. In contrast, the kinetics and pharmacology of a receptor prepared from mammalian brain are consistent with what one might expect of a taurine-specific receptor, i.e., the binding of ^3^H-taurine was highly specific, and not affected by agonists or antagonists of receptors for glutamate, glycine, benzodiazepine, and GABA_B_, nor by monovalent or divalent cations [154]. The binding was completely abolished by 0.1 mM cobalt, zinc, or mercury, suggesting the presence of free sulfhydryl groups near or at the ligand-binding site.

Another study examining proteins that interact with taurine used the cross-linker bis-(sulfosuccinimidyl) suberate (BS3) to covalently bind ^3^H-taurine to cell surface proteins on membranes from the olfactory organ of the spiny lobster [155]. In their inhibition studies, only taurine inhibited the crosslinkage of ^3^H-taurine to the membrane, and the taurine-evoked behavioral search response was significantly reduced following treatment of their antennules with BS3 + taurine as compared with animals treated with BS3 alone. This suggests that the taurine-labeled binding proteins include taurine receptor proteins involved in the first stage of olfactory transduction. However, neither of these studies attempted to determine the molecular structure of a taurine receptor at the respective sites.

Currently, perhaps the best hope for establishing the molecular structure of a taurine receptor stems from the elegant work of Anderson and Trapido-Rosenthal [156], who discovered a unique taurine receptor candidate at a fast *excitatory* synapse in the motor nerve net (MNN) of the jellyfish *Cyanea capillata*. Intracellular recording from these relatively large cells in the MNN showed that only taurine (a β-sulfonic acid) and β-alanine (a β-carboxylic acid), both of which are present in the neurons and released on depolarization, produced responses consistent with those of the normal excitatory post-synaptic potentials (EPSPs) in these cells. They tested the effects of 28 candidate neurotransmitters including glycine, GABA, dopamine, epinephrine, acetylcholine, and a variety of neuropeptides and nucleotides. Although a very small response was elicited with GABA, the most effective agents were taurine and β-alanine, both of which produced large depolarizations that varied in amplitude with membrane potential. Either or both of these amino acids, or a closely related unidentified compound, is likely to be the neurotransmitter at this fast chemical synapse. The magnitude of the changes they elicited was exceeded only by the taurine analog homotaurine (3-aminopropane-sulfonic acid), although the time course of the response decay was much slower. As the authors noted, while it is evident that glycine is not a transmitter at the MNN synapse, the features of the taurine response are unlike that typically seen in mammalian preparations, i.e., a hyperpolarizing, inhibitory response. The slow, long-lasting nature of these depolarizing responses suggests that they may be mediated by metabotropic receptors rather than the ionotropic receptors acting at the fast excitatory synapses of the MNN. It remains to be seen whether cloning and expression of the proteins of the MNN neurons will yield a taurine-specific receptor.

### Summary and Final Thoughts

In this brief review, we have described several conditions, both normal and pathological, **i**n which taurine has been shown to exert a significant effect. More inclusive reviews can be found in excellent accounts by Huxtable [157], Lombardini [158], Timbrell et al. [159], Schaffer et al. [134], and Yamori et al. [34]. In addition, the reader may wish to consult the many insightful studies on the effects of taurine on intercellular communication (cf. [160-163]), the axonal transport of taurine in the retina and CNS [25,164-166], and a comprehensive review devoted solely to the actions of taurine in the retina [167].

Taurine plays an important role as a basic factor for maintaining cellular integrity in the heart, muscle, retina, and throughout the CNS. As we have attempted to show, this ubiquitous amino acid is a potent cytoprotective agent; moreover, it is considered to be a neurotransmitter candidate, is clearly a modulator of neuronal activity, and is a molecule that deserves significantly more attention than it has received thus far. It is likely that the multiple functions of taurine we have described are mediated at different loci on both extracellular sites (e.g., to participate in neuronal activity, stimulate rod production) and intracellular targets (e.g., to fulfill its role in development and cytoprotection).

Although there is considerable evidence that, in specific circumstances, taurine can interact with GABA_B_ receptors to activate a metabotropic pathway, neither the intracellular link nor a taurine-specific receptor has yet to be identified at the molecular level. However, the quest may end before too long. Although there is no shortage of nonhuman neuronal systems in which taurine is a prominent component, e.g., the squid giant axon [168], the mollusk *Aplysia *[169], and the migratory locust [170], results obtained from the jellyfish motor nerve net suggest that a taurine-specific receptor may be present in this unusual beast [156]. If it could be described at the molecular level, this would be a major achievement, and a significant step toward unraveling the pathway(s) by which taurine provides cytoprotection, osmoregulation, neuromodulation, and the myriad of important functions it serves in humans and animals.

In the preface to the second edition of their fine text on *Molecular Cell Biology* [171], James Darnell, Harvey Lodish, and David Baltimore state that the quest in all biologic disciplines is the same: “to discover proteins that could carry out specific biologically important tasks.” A rephrasing of that statement might well include all molecules that engage in such tasks, even the “nonessential” amino acid taurine, which participates in so many vital biological functions.
